# Median Nerve Compression by Hematoma Due to an Iatrogenic Pseudoaneurysm of the Radial Artery

**DOI:** 10.1097/GOX.0000000000007049

**Published:** 2025-08-12

**Authors:** Johannes C. Heinzel, Henrik Lauer, Jonas Kolbenschlag, Adrien Daigeler, Michael R. Bauer

**Affiliations:** From the Department of Hand-, Plastic, Reconstructive and Burn Surgery, BG Unfallklinik Tuebingen, University of Tuebingen, Tuebingen, Germany.

## Abstract

In this article, we report a case of severe median nerve compression in the forearm caused by a large hematoma resulting from an iatrogenic pseudoaneurysm of the radial artery. Two weeks before presenting to our emergency room with neuropathic pain and persistent numbness of the palm, thumb, index, and middle fingers, as well as thumb motor deficits, the patient had undergone wrist denervation at an external outpatient clinic. Duplex ultrasound indicated a large pseudoaneurysm of the radial artery, which was confirmed by computed tomography angiography. The patient underwent emergency surgery, including evacuation of the hematoma compressing the median nerve beneath the forearm fascia, repair of the injured radial artery, and decompression of the median nerve. The procedure also included a carpal tunnel release. The patient experienced symptom improvement immediately after surgery. By the third postoperative day, when discharged, sensation in the median nerve distribution was equal bilaterally as assessed by the TEN-test, and he was able to abduct his thumb using the abductor pollicis brevis muscle. This article highlighted a relatively rare complication following wrist denervation surgery. Because this procedure involves exposure of the radial artery, there is an increased risk for iatrogenic pseudoaneurysm. This case also illustrated the diagnostic steps and microsurgical expertise required to manage such complications and to prevent severe damage to the median nerve through early decompression.

Although nontraumatic compression neuropathies—such as carpal tunnel syndrome, the most common, and cubital tunnel syndrome—are frequently encountered, nerve compressions due to trauma are less common.^1^ Among these, nerve compressions caused by arterial pseudoaneurysms resulting from iatrogenic injuries following catheterization or other surgical procedures are even rarer, though reported by several authors.^2,3^ Interestingly, although such reports most commonly involve pseudoaneurysms of the brachial artery, to the best of our knowledge, a pseudoaneurysm of the radial artery has been reported only once as a cause of median nerve compression. In that case report, Bauer et al^4^ described a case of carpal tunnel syndrome developing 18 months after a transradial coronary intervention, caused by a pseudoaneurysm of the radial artery accompanied by an arteriovenous fistula.

Many commonly performed procedures on the hand and forearm, such as wrist denervation surgery (ie, resection of the afferent articular branches to the wrist supplied by the median, ulnar, and radial nerves^5^) for chronic wrist pain, carry the risk of iatrogenic vascular injury.^6^ However, no case of median nerve compression following an iatrogenic radial artery injury during wrist denervation surgery has been reported to date. In this case report, we present the clinical course of a patient who presented to our emergency department with symptoms of acute median nerve compression approximately 2 weeks after undergoing wrist denervation surgery in an outpatient setting.

## CASE REPORT

We describe the case of a 43-year-old man who underwent left wrist denervation surgery for chronic wrist pain at an external outpatient clinic. Thirteen days postoperatively, he presented to our emergency department complaining of persistent numbness in the left palm, thumb, index, and middle fingers, as well as motor deficits in his left thumb. These symptoms began approximately 8 hours after the surgery. He also reported severe neuropathic pain in the same regions, which had started approximately 24 hours before presenting.

**Fig. 1.**
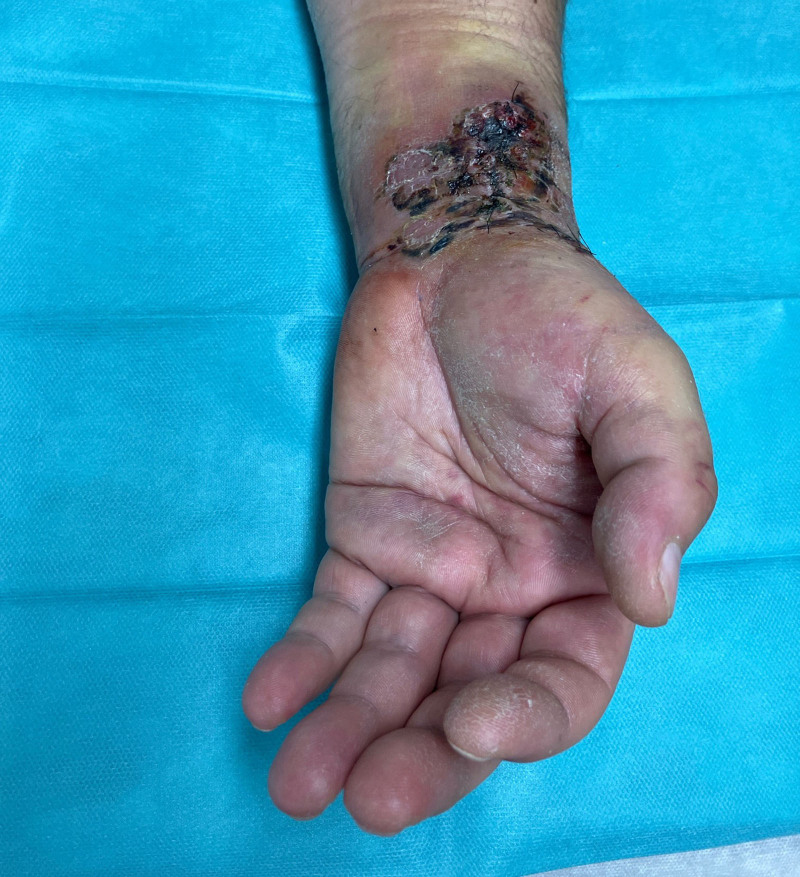
Preoperative image of the patient’s left wrist at presentation to the emergency room. Note the prominent swelling and superficial skin necroses in the wrist area.

**Fig. 2.**
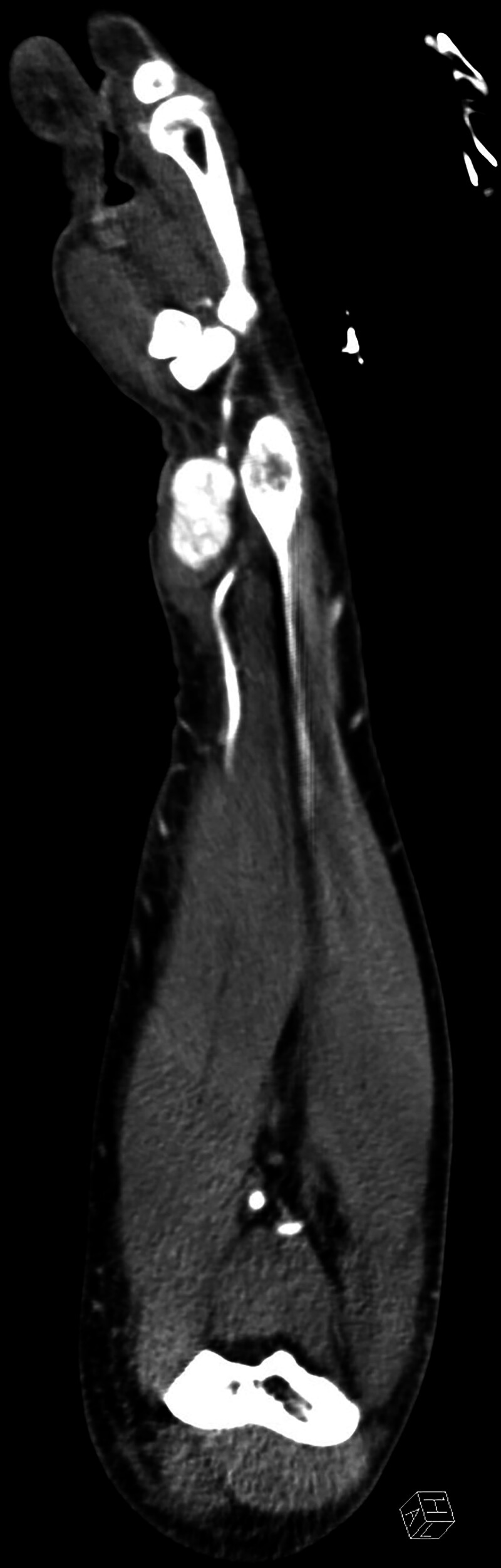
Computed tomography angiography revealing a pseudoaneurysm of the radial artery as the underlying cause of median nerve compression by the resulting large hematoma.

On inspection, there was significant swelling of the left wrist along the course of the radial artery (Fig. 1). Clinical examination revealed anesthesia in the median nerve distribution of the left hand and paresis of the abductor pollicis brevis muscle (strength grade M1, Medical Research Council scale). A visible, pulse-synchronous pulsation of the wrist mass prompted a duplex ultrasound examination, which indicated a large pseudoaneurysm of the radial artery and a hematoma tracking beneath the antebrachial fascia, compressing the median nerve. An emergency angio-computed tomography scan (Fig. 2) confirmed a 30 × 15 × 15 mm pseudoaneurysm of the radial artery.

**Fig. 3.**
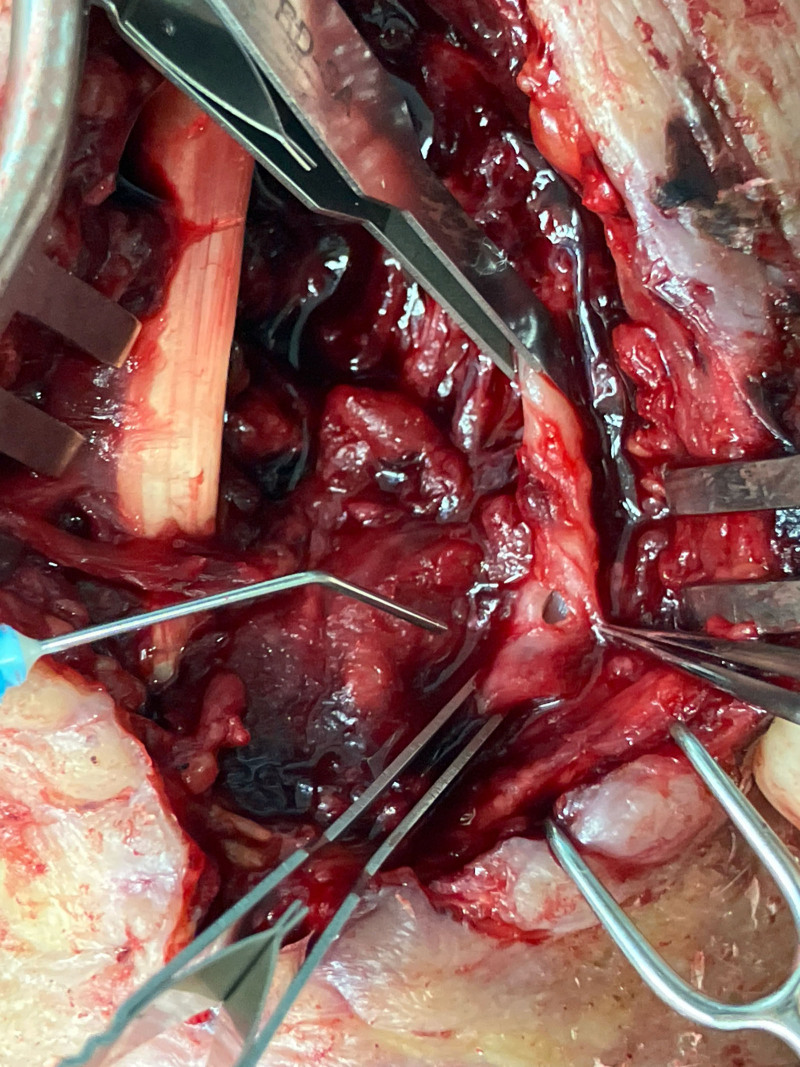
Iatrogenic injury of the left radial artery.

**Fig. 4.**
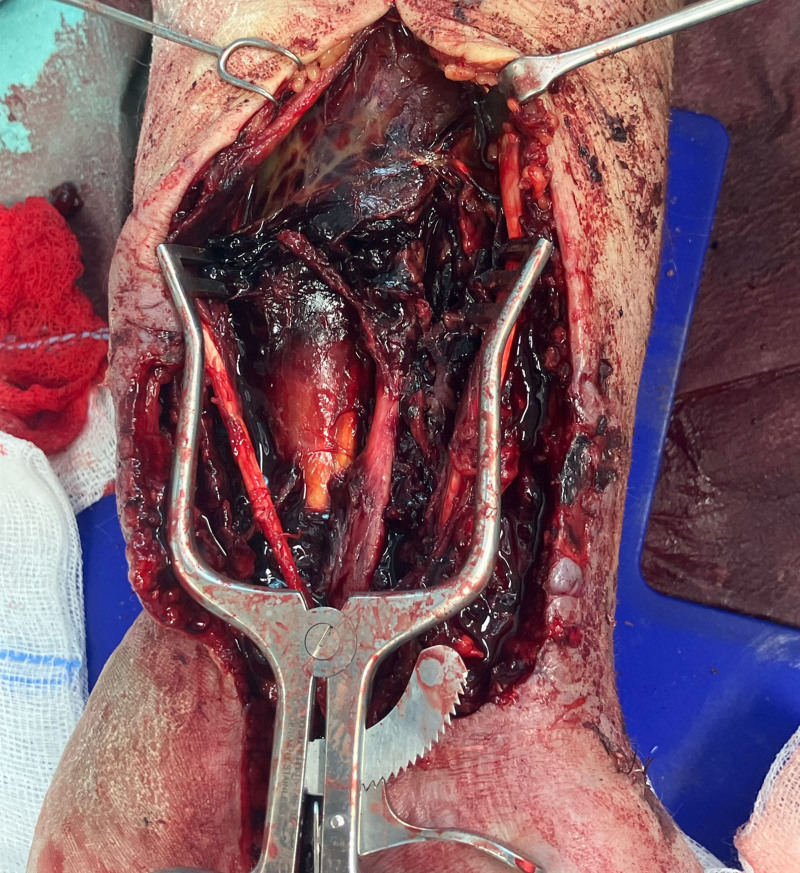
Large hematoma in the left forearm compressing the median nerve beneath the forearm fascia.

The patient underwent emergency surgery. The hematoma was evacuated, revealing an iatrogenic injury to the radial artery as the source of the pseudoaneurysm (Fig. 3). The artery was repaired with 10-0 microsutures without the need for a vein patch or graft. The median nerve was found to be in continuity, except for a small fascicle, likely the palmar branch, which had been previously severed. The nerve was severely compressed beneath the forearm fascia by the hematoma (Fig. 4). Decompression and neurolysis of the median nerve were performed along with a carpal tunnel release. Superficial skin necroses were debrided but required no additional surgical treatment.

Postoperatively, the patient experienced immediate and steady improvement. Within 48 hours, he regained sensation in the thumb, index, and middle fingers and was able to abduct his thumb using the abductor pollicis brevis. He was discharged 3 days postoperatively and reported normal sensation in the median nerve distribution, with symmetrical results confirmed by the TEN-test. He could abduct his thumb against moderate resistance, indicating rapid motor recovery of the median nerve following hematoma evacuation and decompression.

## DISCUSSION

Our case report emphasized a relatively rare complication following wrist denervation surgery—namely, pseudoaneurysm of the radial artery with subsequent median nerve compression beneath the forearm fascia by the resulting hematoma, requiring emergency nerve decompression and arterial repair in the forearm. Although median nerve compression by iatrogenic pseudoaneurysms of the brachial artery has been reported following minimally invasive procedures,^7^ the occurrence of such a complication after open surgery, such as wrist denervation, is rare but significant and must be addressed promptly.

Patient-related risk factors for arterial pseudoaneurysms include the use of anticoagulants and antiplatelet agents, hypertension, and age older than 65 years.^8^ Although these factors were absent in our case, awareness of them can help identify at-risk patient populations and aid in the prevention of complications such as the one described here.

Fortunately, in our patient, all symptoms resolved rapidly and completely. Therefore, the median nerve lesion can be classified as neurapraxia.^9^ Because nerve recovery after acute compression in the upper extremity can take several weeks to months,^10^ the degree of nerve injury in our case can be considered mild. However, it is likely that continued or chronic compression would have led to a higher grade lesion, making full functional recovery less probable over time. Although a preoperative duplex ultrasound was sufficient to identify the underlying cause, computed tomography angiography provided valuable information regarding the pseudoaneurysm’s exact dimensions and supported preoperative surgical planning. Our case also underscores the importance of microsurgical expertise in managing complications that can arise from commonly performed hand procedures such as wrist denervation.

## DISCLOSURE

The authors have no financial interest to declare in relation to the content of this article.

## ACKNOWLEDGMENTS

The authors acknowledge support from the Open Access Publishing Fund of the University of Tuebingen. We also acknowledge support from Lippincott Williams & Wilkins Editing Services for improving the grammar and readability of the article. This was provided as a paid service.

## DECLARATION OF HELSINKI

This study was conducted in accordance with the ethical standards of the institutional and/or national research committee and with the 1964 Declaration of Helsinki and its later amendments or comparable ethical standards.
